# Antipsychotics, Metabolic Adverse Effects, and Cognitive Function in Schizophrenia

**DOI:** 10.3389/fpsyt.2018.00622

**Published:** 2018-12-05

**Authors:** Nicole E. MacKenzie, Chantel Kowalchuk, Sri Mahavir Agarwal, Kenya A. Costa-Dookhan, Fernando Caravaggio, Philip Gerretsen, Araba Chintoh, Gary J. Remington, Valerie H. Taylor, Daniel J. Müeller, Ariel Graff-Guerrero, Margaret K. Hahn

**Affiliations:** ^1^Centre for Addiction and Mental Health, Toronto, ON, Canada; ^2^Institute of Medical Science, Faculty of Medicine, University of Toronto, Toronto, ON, Canada; ^3^Department of Psychiatry, University of Toronto, Toronto, ON, Canada; ^4^Women's College Hospital, Toronto, ON, Canada

**Keywords:** schizophrenia, antipsychotics, metabolic syndrome, cognitive dysfunction, mechanism, inter-relationship

## Abstract

Cognitive impairment is a core symptom domain of schizophrenia. The effect of antipsychotics, the cornerstone of treatment in schizophrenia, on this domain is not fully clear. There is some evidence suggesting that antipsychotics may partially improve cognitive function, and that this improvement may vary depending on the specific cognitive domain. However, this research is confounded by various factors, such as age, duration/stage of illness, medication adherence, and extrapyramidal side effects that complicate the relationship between antipsychotics and cognitive improvement. Furthermore, antipsychotics—particularly the second generation, or “atypical” antipsychotics—can induce serious metabolic side effects, such as obesity, dyslipidemia and type 2 diabetes, illnesses which themselves have been linked to impairments in cognition. Thus, the inter-relationships between cognition and metabolic side effects are complex, and this review aims to examine them in the context of schizophrenia and antipsychotic treatment. The review also speculates on potential mechanisms underlying cognitive functioning and metabolic risk in schizophrenia. We conclude that the available literature examining the inter-section of antipsychotics, cognition, and metabolic effects in schizophrenia is sparse, but suggests a relationship between metabolic comorbidity and worse cognitive function in patients with schizophrenia. Further research is required to determine if there is a causal connection between the well-recognized metabolic adverse effects of antipsychotics and cognitive deficits over the course of the illness of schizophrenia, as well as, to determine underlying mechanisms. In addition, findings from this review highlight the importance of monitoring metabolic disturbances in parallel with cognition, as well as, the importance of interventions to minimize metabolic abnormalities for both physical and cognitive health.

## Introduction

Schizophrenia is a severe psychiatric disorder characterized by a wide range of symptoms. These include positive symptoms, such as hallucinations and delusions, negative symptoms, such as apathy and amotivation, and impaired cognition (1). From the advent of chlorpromazine in the 1950s, the first antipsychotic (AP) for the treatment of schizophrenia, to today, APs uniformly alleviate positive symptoms with minimal effect on negative symptoms (2). Treatment with APs may be associated with a modest positive impact on cognitive functioning but there are many caveats to this finding (3). Furthermore, most APs, led by clozapine and olanzapine, cause serious metabolic side effects including weight gain, insulin resistance, and dyslipidemia. Independent studies suggest that about 50% of patients treated with APs develop metabolic complications (4, 5). These rates are even higher in young, first episode patients (6, 7). Cognitive impairment and metabolic aberrations have important functional and physical consequences. Schizophrenia continues to be associated with severe disability, owing largely to cognitive impairments (2), while metabolic illness contributes to decreased patient lifespan by about 20 years due to cardiovascular disease (8). In addition, these two critical domains of health and functioning might interact, as metabolic dysregulation is associated with impaired cognition in both patients with schizophrenia (9), as well as, the non-psychiatrically ill (10). The inter-relationships between cognition and metabolic side effects are therefore complex, and this review aims to examine them in the context of schizophrenia and AP treatment.

### Cognition and Schizophrenia

Cognition is broadly defined as the ability to accurately perceive, attend to, process and remember information (2). An impairment in cognition is a hallmark symptom experienced by individuals with schizophrenia, and has been postulated to be a core aspect of the disorder (11). Important work over the last couple of decades has informed our understanding of the nature and properties of these deficits and has been extensively reviewed elsewhere (3, 12, 13). The field, however, is characterized by heterogeneous findings and many unanswered questions. This has been fueled, in part, by heterogeneity in the clinical profiles of the participants recruited and the methods used for cognitive testing. However, there are certain broad points of agreement that are briefly summarized below.

Cognitive deficits are observed in the majority of patients with schizophrenia, and this deficit is robust with a large effect size (3, 13). However, inter-subject variability is also an important aspect of this impairment. There is both a generalized impairment of cognition, as well, as impairment in several specific areas of functioning (14). These specific domains include speed of processing, attention, working memory, verbal learning and memory, visual learning and memory, reasoning and problem solving, and social cognition (15). The deficits are present pre-morbidly and tend to persist throughout the course of the illness (12). The course of these deficits is not completely clear, however, but in general have a weak relationship with positive symptoms, and a stronger association with the severity of negative symptoms (16). These impairments are also seen in unaffected relatives, albeit to a lesser degree, suggesting genetic underpinnings and shared risk among family members of schizophrenia patients (17). As will be reviewed, emerging evidence suggests that impairment in cognitive functioning may be exacerbated in the presence of metabolic comorbidity, which may in part be caused by the main-stay treatment for psychosis (i.e., AP medications) (9). Cognitive impairments are of fundamental importance since they substantially change the way individuals interact with their environment and have been linked directly and robustly to social and vocational functioning outcomes (2). This impact on functional outcomes makes it imperative that we understand fully the effect of treatment with AP medications on cognitive functioning. This includes consideration of medication-associated metabolic side effects and whether these may limit treatment efficacy in this critical illness domain.

### Antipsychotics and Cognition

Given that antipsychotics are the cornerstone of schizophrenia treatment, the interaction of APs with cognitive impairment is of critical importance when looking at treatment outcomes. In spite of the large number of studies evaluating this relationship, however, the effects of AP medications on cognition remains controversial (13). Historically, the so-called “first generation” or “conventional” class of APs (FGA) was considered to have neutral or even detrimental effects on cognitive functioning. The introduction of so called “atypical,” or second-generation APs (SGA) spawned hope that these newer medications would improve cognitive functioning relative to their “first generation” counterparts. Indeed, soon after the introduction of the newer APs, several studies seemed to suggest that treatment with these compounds could improve cognitive functioning in schizophrenia patients (18, 19). For example, multiple studies have shown that SGAs improved set shifting ability (1–3), a component of cognitive flexibility. Clozapine has shown large positive effects in attention and verbal fluency with modest improvements in executive functioning (20) and delayed recall (21). Olanzapine has also demonstrated significant improvement in vigilance, selective attention, delayed recall, as well as, verbal learning and memory, verbal fluency, and executive functioning (20, 21). Risperidone has generally shown more modest effects in comparison to the aforementioned medications, demonstrating moderate improvements in working memory, executive functioning, attention (20, 21), and delayed recall (21). Aripiprazole has been shown to improve reaction time with correct responses to stimuli (22), as well as, verbal cognitive functioning (23) while quetiapine has been shown to improve global cognitive functioning in the early stages of treatment (24–26) and verbal short-term memory (27).

While there is strong support for the effect of SGAs and their impact on cognition, more recent work has challenged the finding that they are superior to FGAs in this regard. Results from the Clinical Antipsychotic Trials of Intervention Effectiveness (CATIE) study, which included a large sample size of chronic schizophrenia patients and sample characteristics considered reflective of the general schizophrenia population, indicated that APs are very similar in their action across chemical classes, and this similarity extends to the effects APs have on cognition. Additionally, the effect size for improvement in cognition was found to be small (28), with questionable clinical significance (29). The debatable superiority of SGAs on cognitive functioning was also highlighted by a meta-analysis that suggested that “older” drugs did, in fact, have a moderate beneficial effect on cognition (30). Two recent network based meta-analyses have shed further light on this question (31, 32). When nine studies investigating long-term (>6 months) effect of APs were meta-analyzed, olanzapine and quetiapine emerged superior (32), but when all studies longer than 8 weeks were considered, there were no clear differences between antipsychotics (31). Furthermore, short follow-ups and methodologies that do not allow for a confident discounting of practice effects raise questions over the claim that APs significantly improve cognition, as practice effects may contribute to the modest gains seen across treatments (12, 28).

There are several confounders that likely have a bearing on this relationship including general symptom improvement, the stage, and duration of the illness, dose of the AP, adherence to treatment, medication-related sedation, and anticholinergic side effects. Many of these aspects have been investigated and reviewed in detail elsewhere (33–37), and are summarized briefly here.

#### General Symptom Improvement with AP Treatment in Relation to Cognition

The degree to which general symptom improvement contributes to the changes in cognitive outcomes seen with AP treatment is an important consideration (38). More specifically, it is important to think about how different symptom domains interact (i.e., positive, negative, cognitive) and whether improvement of some symptoms relates to improvement in others. Studies differ with respect to their findings in this area. For example, multiple studies have demonstrated that although positive (and to some degree negative) symptoms may improve with AP use, these changes do not interact with executive functioning (27, 39–41). This also speaks to the notion that certain aspects of cognitive deficits (i.e., cognitive flexibility) may be inherent to the illness of schizophrenia, occurring independently of positive or negative symptomatology (40–42). Conversely, there is also evidence to suggest that improvements in cognition correlate with improvements in symptomology. For example, improvement in processing speed has been found to relate to negative symptom improvement, where improvement in negative symptoms occurred alongside improvements in processing speed after 24 weeks of treatment with a second generation antipsychotic (43). Taken together, the possibility arises that symptoms impact specific facets of cognition, rather than impacting global cognitive function.

#### Illness Course and Stage of Intervention in Relation to Cognitive Improvement

It may also be important to consider the stage at which treatment begins when looking for interactions between improvement in specific symptom domains and subsequent cognitive improvement. A recent review suggests that the small cognitive improvement seen after AP treatment initiation is possibly due to an improvement in psychotic symptoms, and that there is not much further improvement in cognitive outcomes beyond the first 1–2 years of treatment (12). This is in agreement with studies that have evaluated cognition in patients either over the course of illness or at varied time points. In first-episode patients, significant improvement in cognition has been reported across many studies and small but significant cognitive improvements related to AP treatment have been seen in first-episode patients as early as 3 months into treatment, in correlation with positive symptom alleviation (44). Significant improvements also continue to be seen in this population at three (45) and 5-years follow-up (46), supporting the idea that if treatment is adequate in the early stages of psychosis, greater improvements in cognition are evident without later decline (45). These outcomes are different than what has been observed in chronic schizophrenia. Studies in chronically ill patients often show little improvement in cognitive symptoms with AP treatment; one study which looked at patients with chronic schizophrenia found no cognitive improvement at 6-months follow-up, and found that this lack of improvement was independent of positive symptom alleviation, as rated on the Positive and Negative Syndrome Scale (PANSS) (47). Cognitive functioning in the chronic stage of the illness is sometimes described as static, as even at a minimum of a year follow-up, no significant improvements are evident (48). Therefore, it appears that the potential cognitive gains with AP treatment are most prominent during the early stages of the illness.

#### Antipsychotic Dose and Adherence in Relation to Cognitive Improvement

There are several other factors that may impact the extent to which APs augment cognitive outcomes. The dose of AP medication may have an effect on cognition, with higher AP doses, as well as, AP polypharmacy, reported to be associated with worse cognitive functioning (49, 50). Further to this point, it has been reported that when AP dose is reduced, cognition significantly improves in several domains, including memory, visuospatial, language, attention, and delayed memory (51–54). There may also be a relationship between dose and length of time receiving treatment with high vs. low AP dose. One study has shown that with higher doses of APs taken over the long term, verbal learning and recall performance decline significantly over time, independently of age of illness onset or severity of illness. Interestingly, there were no significant differences in cognitive decline observed between low-dose cases and non-psychiatric controls over the 9-years naturalistic follow up (50). Conversely, in a meta-analysis of older schizophrenia patients, medication status or dosing according to chlorpromazine equivalence failed to demonstrate a significant association with cognition over 1–6 years of follow-up (55).

Medication adherence also contributes to the relationship between AP and cognitive functioning. In studies where medication adherence was considered, those who were adherent, not surprisingly, have been reported to show greater improvements in cognition compared to those who did not take their medications regularly (37). However, whether poor adherence leads to a lack of improvement in cognition or inherent cognitive deficits related to the diagnosis of schizophrenia lead to poor medication adherence remains an unanswered question. Further to this point, a recent review has shown that long acting injectable (LAI) agents show superiority in terms of efficacy when compared to oral agents (56), with reported improvement in general cognitive performance in individuals switching from oral to injectable agents (57). In a direct comparison between risperidone in LAI vs. oral form, it was found that white matter and myelination increased with LAI use while it declined in oral risperidone use (58). This was attributed to adherence to medication (58) and suggests that perhaps adherence to treatment may impact the extent to which cognitive benefits are seen.

#### Extrapyramidal Adverse Effects and Cognition

Extrapyramidal symptoms (EPS) represent one of the more well-known side-effects of AP medications. While these side-effects have been found to be correlated with impaired cognitive abilities, such as set-shifting (59), it appears that cognitive impairments may be associated with EPS even in the absence of active antipsychotic treatment (35). This raises the question of whether the apparent impairment in cognition is a result of the medication or inherently linked to the illness and its premorbid cognitive dysfunction. Interestingly, with the introduction of SGAs less focus has been given to EPS and studies generally do not report significant correlations with cognition (60–62).

With this said, in cases where EPS is present, it is also critical to account for the role of anticholinergic treatments adjunct and inherent within antipsychotics when evaluating cognitive outcomes. Recent reviews have found that co-treatment with anticholinergic medications have a negative effect on cognition (36, 63), with individuals with schizophrenia particularly susceptible to these effects (64). The aspects of cognition which are most commonly impaired include memory and attention (64). Of note, cognition improves when anticholinergic treatment is discontinued (36, 63).

While each of the above-mentioned factors are important, another facet of AP effects that has not received adequate attention is the effect of AP-induced metabolic dysfunction on cognition. This presents a potentially modifiable therapeutic target (i.e., metabolic syndrome), the management of which can potentially lead to better clinical outcomes.

### Antipsychotics and Metabolic Dysregulation

Although “atypical” or SGAs, as a class were associated with higher metabolic risk than first-generation antipsychotics, a more nuanced understanding of this “class” [which in itself has become a topic of controversy (65, 66)], has shown that there is a differential metabolic liability [i.e., olanzapine = clozapine > sertindole > risperidone > = quetiapine > = amisulpride > ziprasidone, lurasidone, aripiprazole 33, 67] among the different constituent medications classified as an SGA. More recent work, however, suggests that all AP drugs are associated with early significant weight gain (68), particularly in young AP-naïve patients (69–72). The effect on weight gain is also of FGAs, which have historically been considered to be metabolically neutral; haloperidol has been found to cause significant weight gain in AP-naïve patients, with an average 3.8 kg weight gain within the first 3 months of treatment (70). Interestingly, while long-term prospective trials examining AP-induced weight gain are lacking, it has been demonstrated that when drug-naïve first episode patients are followed over the long-term (>2 years), the differences between individual antipsychotic agents disappear (70). This may suggest that treatments differ more by pattern of weight gain, rather than amount.

Similar to the trends seen with weight gain, studies published recently show concerning rates of prediabetes (>15%) in first episode patients within 6 months of AP exposure (73), and significantly higher cumulative risk and incidence of type two diabetes (T2D) (74). A recent population-wide national registry estimated that APs, irrespective of whether they were FGAs or SGAs increased the risk of diabetes 3-fold in patients with schizophrenia (75). Moreover, while overweight and obesity represent leading risk factors for diabetes, work in animals and humans consistently demonstrates that APs can directly induce insulin resistance and glucose dysregulation even in absence of weight or adiposity changes (76). Notably, initial concern with metabolic side-effects linked to these agents was focused primarily on cardiometabolic morbidity and mortality (5). These concerns have been extended to quality of life, self-esteem, medication adherence (77), and most recently, as will be reviewed in detail, potentially to cognitive functioning.

### Metabolic Dysregulation and Cognition in Non-Psychiatrically ILL Populations

The term metabolic syndrome (MetS) refers to the presence of multiple, interrelated cardiovascular risk factors occurring simultaneously (78, 79). While specific criteria for MetS differ according to different guidelines, in general, three out of the five following criteria must be met: increased waist circumference, elevated triglycerides, reduced high-density lipoprotein (HDL), cholesterol, elevated blood pressure, and elevated fasting glucose (78, 80). There is a large body of data demonstrating impairments in cognition as a result of MetS in psychiatrically non-ill individuals (10, 81–85) and a recent literature review found a strong link between MetS and specific deficits in memory, visuospatial abilities, executive function, processing speed, and intellectual function (86). There are also significant correlations between specific components of MetS and cognition. For example, patients with hypertension performed significantly worse on executive functioning tasks, which was correlated with reduced frontal lobe volume and impaired glucocorticoid feedback (87). Moreover, high body mass index (BMI) has been found to correlate with lower cognition scores (12, 95).

An accumulating body of evidence suggests a mechanistic relationship between cognitive decline, obesity, MetS, diabetes, and Alzheimer's disease (88, 89) with shared neuropathological characteristics and insulin resistance, oxidative stress, and a persistent inflammatory state as the core pathology. These lines of evidence suggest a tight link between cognitive and metabolic processes and outcomes, which would be predicted to extend to patients with schizophrenia as well. If this association does hold in patients with schizophrenia, an additional intriguing question is also the role of AP-related metabolic perturbations in the context of AP-related effects (or lack there-of) on cognitive function. Thus, focus must be turned to: (1) the association between MetS and cognitive deficits in schizophrenia, and, (2) whether AP-related metabolic dysfunction could, in part, mediate this association.

### Metabolic Dysregulation and Cognition in Schizophrenia

MetS occurs in ~33.5% of patients with schizophrenia (90). There are multiple factors which contribute to the elevated prevalence rate of MetS in the schizophrenia population, including lifestyle factors, such as smoking (91), poor eating habits and sedentary behavior (see Ringen (92) for an in-depth review). Biological and genetic factors inherent to the illness of schizophrenia and treatment response also overlap with genes related to metabolic function (93). For example, genes associated with the illness of schizophrenia have independently been linked to regulation of fat mass, leptin signaling (94), insulin signaling, glucose metabolism, and inflammation (95, 96). In addition, as reviewed here, antipsychotic medications are well-established to contribute to cardiometabolic risk in this already metabolically vulnerable population. Some of these agents are also implicated in cognition. The serotonin receptor gene 5HT2A has been found to influence both lipid levels and glucose intolerance (94), as well as, attention span and cognitive flexibility (97). As well, the methylenetetrahydrofolate reductase (MTHFR) gene is believed to increase the susceptibility of developing MetS in patients with schizophrenia (94), and is also implicated in poorer verbal recall and cognitive flexibility (98). Thus, it appears that the genes implicated in poor metabolic outcomes for individuals with schizophrenia could also be associated with cognitive outcomes.

Taken together, it is perhaps not surprising that the majority of studies in schizophrenia suggest that the relationship between MetS and cognitive dysfunction is similar among schizophrenia patients as it is to non-psychiatrically ill individuals. MetS has a negative impact on cognition, and indeed schizophrenia patients with MetS have been reported to perform worse on measures of cognition than those with schizophrenia in the absence of MetS (9, 99, 100). Performance appears to be negatively impacted on tasks measuring processing speed, memory, attention, and reasoning in those with co-morbid MetS as compared to those with schizophrenia alone (9, 99, 100). Furthermore, this impairment has been shown to develop post-morbidly (101), suggesting that there could be an active mechanism in the developmental course of the disorder that may impair cognition as one's metabolic health declines.

With that said, not all studies have found a relationship between MetS and cognitive impairment in schizophrenia. As reviewed by Bora and colleagues, the CATIE trial stands out as the largest study which failed to find an association (102). Other studies, which may have failed to find an association between MetS and cognitive dysfunction have however found that specific aspects of MetS (i.e., hypertension) were correlated with lower cognitive scores, whereas other factors, such as increased waist circumference and dyslipidemia were less consistently associated with general impairments in cognition in schizophrenia (49, 103). Other factors, which constitute a poor metabolic profile, such as high BMI, have also not consistently been found to predict poor cognitive functioning across several cognitive domains (104). Furthermore, in one study, hyperglycemia was actually found to predict better verbal memory performance (49).

The discrepancy in the schizophrenia literature examining associations between metabolic comorbidity and cognitive deficits could, in part, be attributable to the amalgamation of diverse metabolic aberrations under the common umbrella of MetS (i.e., considering MetS as merely a binary factor) and suggest value in considering cognition alongside individual metabolic symptoms. Indeed, studies that failed to find a relationship between metabolic aberrations and cognitive functioning, such as CATIE study (102), also did not examine effects of individual components of MetS in relation to cognition. This suggests that individual metabolic outcomes might interact independently with cognitive abilities and should be studied as distinct entities to understand the relationship between metabolic and cognitive parameters better (102).

### Disentangling Antipsychotic-Induced Metabolic Dysregulation in Relation to Cognition in Schizophrenia

Cognition is a core area of impairment in schizophrenia and the impact of APs on cognition is not fully clear. Also, there appear to be associations between cognition and metabolic parameters that are being gradually understood. In summary, independent relationships exist between APs, metabolic outcomes, and cognition: (i) APs might influence cognition (with benefits more consistently seen early on in illness, possibly in relation to improvements in other domains of psychopathology); (ii) APs cause metabolic dysregulation, and (iii) metabolic dysregulation has been linked to cognitive dysfunction, with the predominance of evidence suggesting this association applies to patients with SCZ. However, the majority of existing studies have looked at individual associations between these factors, leaving more complex interactions between these factors largely unexplored. Here, we attempt to identify and extrapolate associations between these three factors in the presence of schizophrenia.

There have been a small number of studies investigating the interaction of APs, metabolic factors, and cognition in schizophrenia (Supplementary Table 1). A study by Chen and colleagues examined metabolic side effects and cognition in first-episode patients to establish whether a cognitive impairment related to metabolic dysfunction would be observed in the early stages of illness and AP treatment (105). In first-episode, newly medicated patients, there was a generally worse metabolic profile and poorer cognitive performance compared to their healthy counterparts; however, the effect size was small. Chen et al. also demonstrated that an inverse relationship between cognition and metabolic symptoms within the newly medicated patients was present but that it was relatively small (105). The specific contribution of AP medications on metabolic comorbidity in relation to cognition was not examined in this cross-sectional design. In another study by Li and colleagues (99), cognitive performance of two groups of chronic schizophrenia patients, according to presence or absence of MetS was cross-sectionally compared, with secondary analyses considering relationships with disease course and antipsychotic medications. In the MetS group, cognition was significantly worse, as has been replicated in similar studies examining the interaction between APs, cognition, and metabolic outcomes (9). In the study by Li and colleagues, this association was attributed to higher fasting triglycerides and systolic blood pressure. Interestingly, however, the course of disease was significantly longer in the MetS compared to non-MetS group. Both duration of disease and use of FGAs (as compared to SGAs) were independently correlated with lower cognitive scores. The caveat here was that the most commonly prescribed FGA was chlorpromazine, which has been established to have a similar metabolic risk profile to higher metabolic liability SGAs, such as clozapine. Thus, while interpretation of specific contributing effects of APs according to predicted metabolic liability was not possible, duration of illness leading to cumulative effects of metabolic comorbidity appeared to emerge as a key predictive factor for worse cognitive function. Similar results were also seen in a study by Boyer et al, who found that longer disease duration was correlated with increased rates of MetS, and this was subsequently correlated with worse cognitive impairment, which was particularly robust in individuals taking second generation APs (106). Taken together, illness duration appears to be the more consistent factor linked to cognitive impairments.

Furthermore, specific components of MetS also appear to be differentially related to cognition in studies which considered the presence of AP medications. Hypertension has been shown to correlate with lower verbal cognition scores, including memory and fluency (49). Triglycerides have also been shown to correlate with poorer cognition scores (100), however this effect has been shown to cease to exist when cholesterol is controlled for (107). As for waist circumference (i.e., abdominal obesity), there is discord in the literature on whether it has an effect on cognition (100, 104) or if it does not (49).

From the evidence presented above, perhaps as illness course and treatment continues metabolic comorbidities accumulate and become more severe (in association with illness duration which is invariably linked in the majority of cases with longer exposure to medications); we subsequently see cumulative effects play out on cognition more clearly. This may explain why the relationship between cognitive performance and metabolic indicators appears to be more robust in patients with longer duration of illness. The accretion of structural changes in the brain (with illness progression, treatment, and metabolic abnormalities) may also contribute to this relationship, as cognitive performance has been shown to be associated with lower gray matter volume (108, 109).

### Potential Mechanisms Underlying Cognitive Functioning and Metabolic Risk in Schizophrenia

While there is a paucity of literature examining the interrelation among APs, cognition, and metabolic disturbances in schizophrenia, the existing literature points to a number of potential mechanistic influences to explain the effects of APs on cognition and metabolic disturbances.

i. Direct receptor action: While all antipsychotics bind to the dopamine, D_2_ receptor, a majority of them bind to multiple other receptors including the histamine, serotonin, muscarinic and adrenergic receptors. Antagonism of each these receptor systems is independently known to affect cognition, as well as, metabolic outcomes, such as weight gain, insulin and glucose dysregulation, and dyslipidemia. Hence, it is quite plausible that the effects of APs on cognition and metabolic measures are really two sides of the same coin: as binding to a given receptor leads to downstream effects on both domains (110). For instance, acute dopamine depletion in humans and rodents reduces peripheral insulin sensitivity (111, 112), purportedly via central dopamine effects in the striatum. Moreover, reduced insulin sensitivity and obesity are associated with reduced dopamine synthesis capacity and endogenous dopamine levels in the striatum (112–114). These neurochemical alterations in turn may be associated with poor cognition. Thus, it is plausible that central dopamine receptor blockade by antipsychotics in schizophrenia may produce both peripheral insulin resistance and poor cognition. Interestingly, evidence suggests that acute antipsychotic exposure does not alter striatal dopamine levels and dopamine synthesis capacity in first-episode patients with schizophrenia (115). While highly speculative, this high dopaminergic reserve in the first-episode may help potentially explain why metabolic side effects have not been related to cognitive deficits in first-episode patients.
ii. Gut microbiome mediated interactions: In addition to the mechanisms of direct receptor action, the role of the gut microbiome (GMB) represents another pathway that may mediate the relationship between cognitive and metabolic dysfunction in schizophrenia (116). The GMB is the collective term for the community of microorganisms residing in the digestive tract. High throughput sequencing is beginning to provide insight into the differences in GMB composition among those with and without schizophrenia. An increased alpha diversity in the GMB has been reported in patients with schizophrenia and has been found to be a significant predictor of schizophrenia status (117). Within the GMB of patients with schizophrenia, most microbial taxa are derived from bacteria and arachae (117). At the phylum level, individuals with schizophrenia appear to have higher proportions of Firmicutes and lower proportions of Bacteroidetes and Actinobacteria in comparison to healthy controls (118). Overall, individuals with schizophrenia have been found to have greater species diversity than those without (119). The severity of different symptom domains of schizophrenia has been found to be significantly correlated with increases in *Lactobacillus* (120). When looking solely at the interplay between the GMB and schizophrenia, it appears that abnormalities in the GMB contribute to the production of key molecules (such as Brain-derived neurotrophic factor) and/or the promotion of an intestinal immune response (120). Both human and mice studies have confirmed that an altered composition of the GMB influences adverse cognitive and metabolic changes seen in schizophrenia (121–123). The relationship between the GMB and schizophrenia pathogenesis becomes even more complicated when antipsychotics are introduced. There are several overlapping sites of action among the GMB and APs (124), and some APs have been shown to modulate the composition of the GMB, which incidentally has been shown to cause weight gain (125). The interactions between schizophrenia, APs, and the GMB have gained increasing attention over the past few years, however to date only one study has investigated the use of antipsychotic and their effect on the GMB in humans (125). This lack of knowledge represents an opportunity to better understand the pathophysiology of schizophrenia concurrently to metabolic comorbidity associated with the GMB and may lead to new therapeutic interventions to target both psychopathological and metabolic indices.
iii. Central insulin resistance: While receptor action and gut microbiome mediated interactions may underlie these relationships, central insulin resistance might be the final common pathway by which metabolic and cognitive outcomes interact. Central insulin has been well-established to regulate cognition in humans. Intranasal insulin, which delivers insulin directly to the CNS, is demonstrated to enhance declarative and working memory (126). Thus, dysregulation or resistance to central insulin action could result in impaired cognition, and indeed, recent work suggests that insulin is the common link between metabolic disorders and disorders of cognition (127). Central insulin resistance has been found to be a central piece of the pathology in cognitive dysfunction in the context obesity and type 2 diabetes, as well as, in aging and dementia. Insulin resistance has been shown to be associated with impaired hippocampal synaptic plasticity and memory (128), as well as, neurogenesis (129). It is possible that this involves the palmitic acid pathway (128). Furthermore, addressing this pathology via administration of insulin intranasally is emerging as a possible treatment strategy in Alzheimer's dementia (130). Interestingly, work from our lab demonstrates that short term use of APs can induce central insulin resistance resulting in glucose dysregulation and changes in feeding independently of weight gain and other longer term metabolic dysfunction (131). The propensity of APs to cause insulin resistance both in the short term, and in the long term via changes in weight and adiposity, might explain, at least in part, worsening of cognition seen in schizophrenia patients with long standing illness who accumulate metabolic dysfunction over time.
iv. Microangiopathy: Another mechanism by which APs, metabolic abnormalities and cognition might interact is microangiopathy or microvascular abnormalities. Microangiopathy is a well-known consequence of metabolic abnormalities including diabetes and metabolic syndrome (132, 133), and is closely related with the development of insulin resistance (134). While classical targets include the eye, the kidney, and the peripheral nervous system, more recent work shows that the effect is more widespread and includes the brain and adipose tissue as well (132). Microangiopathic changes in the brain have been implicated in cognitive decline (135). Furthermore, there is preliminary evidence that APs may affect the neurovascular unit and small vessels in frontal cortex in a post-mortem study (136). Thus, it is possible that microangiopathy could be a potential mechanism by which APs, metabolic abnormalities and cognition interact. The gradual development of microangiopathy would also, in part, explain why the relationship between metabolic abnormalities and cognitive decline is seen later on in the illness course.

Thus, the effects of APs on cognition and metabolic outcomes likely result from a complex relationship between differential receptor binding, changes in glucose metabolism, insulin sensitivity, the gut microbiome, blood vessel related changes, along with other yet to be described mechanisms to manifest in the clinical outcomes seen. This complex interaction, combined with underlying pathophysiological and lifestyle factors, is likely responsible for the heterogeneity in results when addressing the relationship between APs, cognition, and metabolic disturbances.

### Concluding Thoughts

There is still a great deal of uncertainty regarding the relationship between cognition, APs, MetS and schizophrenia. First, there are varying results regarding whether APs actually improve cognition, which aspects of cognition are improved, and the degree of improvement. APs are shown to cause metabolic dysregulation that has been shown to negatively impact cognition. It is tempting to simplify the scenario and assume that as APs induce metabolic dysfunction, cognition should therefore be impaired. However, this does not seem to consistently be the case, given that in some cases, though metabolic side effects were observed, this was not associated with impairments in cognition. As a case in point, young, AP-naïve individuals are most vulnerable to obesogenic effects of AP medications, yet, as reviewed here, the association between MetS and cognitive deficits appears to be most prominent in individuals in the chronic stage of illness. Moreover, it might be expected that individual AP agents that are associated with the greatest metabolic risk (i.e., olanzapine and clozapine), should be associated with worse cognitive functioning. Again, this does not appear to be the case. At first glance these observations would appear to argue against a direct association between AP-induced metabolic adverse effects and detrimental cognitive effects. However, several other points warrant consideration. Firstly, early weight gain, glucose, and lipid abnormalities induced by AP medications may not, in themselves, be sufficient to immediately translate to cognitive impairments. It is possible then that adverse metabolic side-effects of APs act as a catalyst for later development of microvascular insults to the brain that may take place several years following onset of individual metabolic comorbidities. It is perhaps also not surprising that a “between agent,” “according to metabolic liability” effect on cognition is not observed. As discussed in earlier sections, the between agent difference in metabolic liability appears to diminish overtime. Thus, it can be argued that individual differences between agents may be relatively less important than the acceleration in metabolic risk that occurs overtime.

To summarize, the relationship between metabolic comorbidity and worse cognitive function appears to be generalizable to patients with schizophrenia. The current literature, however, does not allow assessment and establishment of a causal connection between the well-recognized metabolic adverse effects of APs and cognitive deficits. A proposed threshold effect in the relationship between metabolic dysfunction and cognition could suggest that earlier on in treatment, metabolic adverse effects related to treatment may have a minimal impact on cognition. However, once an individual passes a certain threshold of metabolic dysregulation, they likely become susceptible to cognitive impairment. Thus, the impact of metabolic side effects may begin to override any positive or neutral effects on cognition observed in individual taking APs once this threshold (which may be dependent on many factors) is reached. Brains of schizophrenia patients show evidence of accelerated aging even early on in the illness (123). Thus, metabolic aberrations may interact with and add to the existing deficits, and thereby contribute to cognitive impairment, with brain aging perhaps playing a role. Like many psychiatric disorders, schizophrenia has been hypothesized to be a syndrome of accelerated aging (137, 138). Support for this stems from the shared syndromes and risk factors between schizophrenia and age related cognitive and metabolic chronic diseases, such as Alzheimer's Disease and Type II diabetes, respectively. Patients with schizophrenia have an increased risk of diabetes and experience an earlier onset of this illness (139). While the focus has been on AP-related metabolic dysregulation, we are reminded that lifestyle factors (smoking, dietary habits, activity levels) also play a critical role in accumulation of metabolic risk in severe mental illness, and both the primary disorder, as well as, accumulation of metabolic risk has been shown to accelerate aging related changes (140, 141).

Going forward, there are important therapeutic implications to keep in mind in regards metabolic disturbances and cognition. Lifestyle interventions, such as diet and exercise, to prevent and/or correct metabolic abnormalities (whether due to APs, lifestyle or illness pathophysiology) can have gains above and beyond physical health. In particular, the available literature suggests that improvement of metabolic status may improve or prevent further impairment of cognition in patients with schizophrenia. For example, a recent review by Firth et al. (142) found that aerobic exercise improved cognitive functioning in patients in schizophrenia (104). In addition, reduction/prevention of AP-induced metabolic side effects is particularly important in the AP-naive and youth population, as APs appear to have the greatest potential to improve cognition at the beginning of treatment, yet AP-naive population are at greatest risk of AP-induced metabolic side effects. This review highlights that assessment of metabolic factors should be considered when studying cognition in schizophrenia, as these metabolic disturbances may be influencing cognitive outcomes. Future studies investigating the mechanism behind the impact of metabolic disturbances upon cognition are suggested to allow for more specific treatments. Overall, more research is required to identify the complex interactions between APs, metabolic status, and cognition, in schizophrenia to enable patients to receive the maximum therapies for cognitive improvements.

## Author Contributions

NM, CK, SA, and MH contributed to the conception and design of the review. NM, CK, SA, and MH wrote the first draft of the manuscript. KC-D, FC, PG, GR, VT, DM, AG-G, and AC wrote sections of the manuscript. All authors contributed to manuscript revision, read and approved the submitted version.

### Conflict of Interest Statement

The authors declare that the research was conducted in the absence of any commercial or financial relationships that could be construed as a potential conflict of interest.
