# Supplementary material for: Antipsychotics, Metabolic Adverse Effects, and Cognitive Function in Schizophrenia
Source: Front Psychiatry. 2018 Dec 5;9:622. doi: 10.3389/fpsyt.2018.00622 (PMC6290646; doi:10.3389/fpsyt.2018.00622)
Supplement: Supplementary file 1 [file Table_1.docx]

Table 1.

*Summary of studies investigating the interaction of APs, metabolic factors, and cognition in schizophrenia*

| Study | Design | Cases | Dx | First-Episode | Mean Age (years) | Cognition | | Metabolic Measures | AP | Results |
| --- | --- | --- | --- | --- | --- | --- | --- | --- | --- | --- |
|  |  |  |  |  |  | Aspects | Assessment |  |  |  |
| Bora et al., 2017 | SR | N/A | SCZ | N/A | 41.3 | Memory, attention, processing speed, verbal memory, EF | MCCB-MATRICS, BACS, TMT, WCST, RBANS, CPT, WMS, ToL, Stroop, Category Fluency, Visual Memory, Verbal Memory, Verbal Learning, Verbal fluency, List Learning, LNS, Digit sequencing, Digit Symbol, D2 attention | MetS (hypertension, dyslipidemia, abdominal obesity, diabetes) | FGAs  SGAs* | - SCZ+MetS performed worse on memory, attention, processing speed, and executive function (compared to SCZ alone) - SCZ+DM performed worse on memory and processing speed - SCZ+obesity was related to worse cognitive impairment (than SCZ alone) - SCZ+dyslipidemia performed worse on EF, verbal memory and attention (than SCZ alone) - All factors of MetS were positively associated to cognitive impairment |
| Boyer et al., 2013 | CSS | 168 | SCZ | No | 36.6 | Memory, attention, reactive flexibility, inhibition capacity, spontaneous flexibility, updating | CVLT, D2 attention task, TMT A+B, Stroop, verbal fluency test, WAIS-III | Waist circumference, triglycerides, HDL, fasting glucose, blood pressure, C-reactive protein | SGAs:  86%  FGAs:  14% | - MetS and inflammation not significantly associated with cognitive performance - Number of MetS criteria associated with lower cognitive performance for CVLT, D2 attention and TMT - Inflammation was not associated with cognitive performance - High triglycerides and abdominal obesity were associated with impaired cognitive performance |
| Depp et al., 2014 | CSS | 804 | SCZ, BP | No | 50.1 | Verbal memory, processing speed, switching, working memory, verbal fluency, problem-solving, sustained attention | RAVLT, TMT A+B, WAIS-III Digit Symbol, LNS, Animal Fluency, WCST-PEN, CPT, Identical Pairs vD | BMI, use of blood pressure or diabetes medication | SCZ: FGAs:  4%  SGAs: 19%  BP:  FGAs: 0.01%  SGAs: 12% | - In the BP group, metabolic parameters were negatively associated with cognitive functioning - In the SCZ group, there were no correlations between metabolic parameters and cognitive functioning |
| Goughari et al., 2015 | CSS | 68 | SCZ, SZA | No | 42.4 | Verbal memory, working memory, motor speed, verbal fluency, reasoning and problem-solving, attention | BACS | Waist circumference, dyslipidemia (HDL-C, triglycerides), blood pressure, fasting glucose | FGAs  SGAs* | - Hypertension predicted poor performance on verbal memory and verbal fluency, while hyperglycemia predicted better scores - Waist circumference and dyslipidemia not significantly correlated with cognitive outcomes - Significant relationship between poorer cognitive performance and higher AP dose |
| Krakowski &  Czobor,  2011 | RCT | 82 | SCZ, SZA | No | 33.8 | Motor function, EF, verbal memory, visual memory | Finger Tapping, Purdue Pegboard Task, WCST, TMT A+B, WMS-R logical memory, immediate and delayed, figural memory, immediate and delayed, WAIS-R Block Design | Cholesterol, glucose, triglycerides, weight | SGAs:  73%  FGAs:  27% | - Increased cholesterol related to improvements in cognition - Triglyceride levels did not interact with cognition when controlled for cholesterol - Glucose and weight were not significantly related to GCI - Cognitive improvement was greatest with OLA compared to CLO or HAL |
| Li et al., 2014 | CS | 388 | SCZ | No | 42.3 | Immediate memory, visuospatial skills, language, attention, delayed memory | RBANS | BMI, fasting blood glucose and lipids, blood pressure, waist-hip ratio, glycol-metabolism and lipo-metabolism tests | SGAs: 79%  FGAs:  21 % | - Significant difference in performance on RBANS, with non-MetS outperforming MetS patients on measures of immediate memory, attention, attention, and delayed memory |
| Lindenmayer et al., 2012 | Post-hoc | 159 | SCZ, SZA | No | 43.5 | Speed of processing, attention/vigilance, working memory, verbal learning, visual learning, reasoning/problem-solving, reading ability | MCCB -MATRICS (minus MSCEIT); WRAT | BMI, fasting blood samples for CBC, SMA 20, including glucose, total cholesterol, total triglycerides, HDL, and LDL | FGAs:  8%  SGAs:  54%  Combo:  39% | - Patients without the MetS performed significantly better on tests measuring processing speed, attention/vigilance, working memory and problem solving/reasoning; no significant difference was noted in processing speed and problem solving/reasoning - Greater waist circumference and triglycerides were associated with lower scores on attention/vigilance, HDL was positively associated with scores on attention/vigilance |

*Legend:*

Dx= Diagnosis, SR= Systematic Review, EF= Executive Functioning Test, SCZ= Schizophrenia, CSS=Cross Sectional Study, BP= Bipolar Disorder, SZA= Schizoaffective Disorder, RCT= Randomize Controlled Trial, MetS= Metabolic Syndrome, HDL= High Density Lipoprotein, LDL= Low density Lipoprotein, BMI=Body Mass Index, CBC= Complete Blood Count, SMA 20= SMAC 20 blood test

*Assessments:*

MCCB-MATRICS= MATRICS Consensus Cognitive Battery, BACS=Brief Assessment of Cognition in Schizophrenia, TMT= Trial Making Test in Schizophrenia, WCST= Wisconsin Card Sorting Test, RBANS= Repeatable Battery for the Assessment of Neuropsychological Status, CPT= Continuous Performance Test, WSM= Wechsler Memory Scale, ToL= Tower of London, LNS=Letter-Number Sequencing, CVLT= California Verbal Learning Test, WAIS-III=Wechsler Adult Intelligence Scale, RAVLT= Rey Auditory Verbal Learning Test, GIC=general cognitive index, WRAT= Wide Range Achievement Test, MSCEIT= Mayer-Salovey-Caruso Emotional Intelligence Test

*Medications:*

AP = Antipsychotic, FGAs= First Generation Antipsychotics, SGAs= Second Generation Antipsychotics, OLA= Olanzapine, CLO=Clozapine, HAL=Haloperidol

*group statistics not indicated
